# Supplementary material for: Structure of the SARS-CoV-2 RNA-dependent RNA polymerase in the presence of favipiravir-RTP
Source: Proc Natl Acad Sci U S A. 2021 Feb 1;118(7):e2021946118. doi: 10.1073/pnas.2021946118 (PMC7896311; doi:10.1073/pnas.2021946118)
Supplement: Supplementary File [file pnas.2021946118.sapp.pdf]

Supplementary information

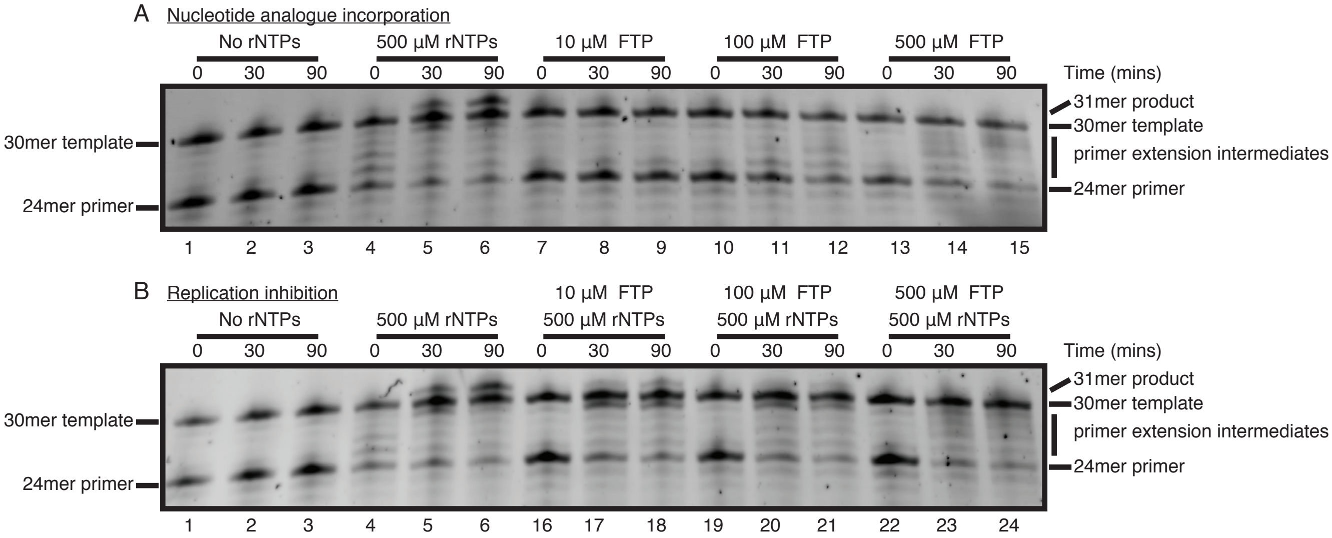

Figure S1: **Reconstitution of SARS-CoV-2 RdRp activity and inhibition by favipiravir-RTP (FTP).** The same assays as in Fig. 1, with the controls on the same gels, are shown. Lane numbering is the same as for Fig. 1. (**A**) Nucleotide analogue incorporation assays compare the efficiency of FTP incorporation, at different concentrations (lanes 7-15), in the absence of rNTPs, into the growing primer strand, to that of rNTPs at 500 µM concentration (lanes 4-6). (**B**) Replication inhibition assays investigate the effect of pre-incubation on the replication reaction (lanes 4-6) with increasing concentrations of FTP for 30 minutes (lanes 16-24).

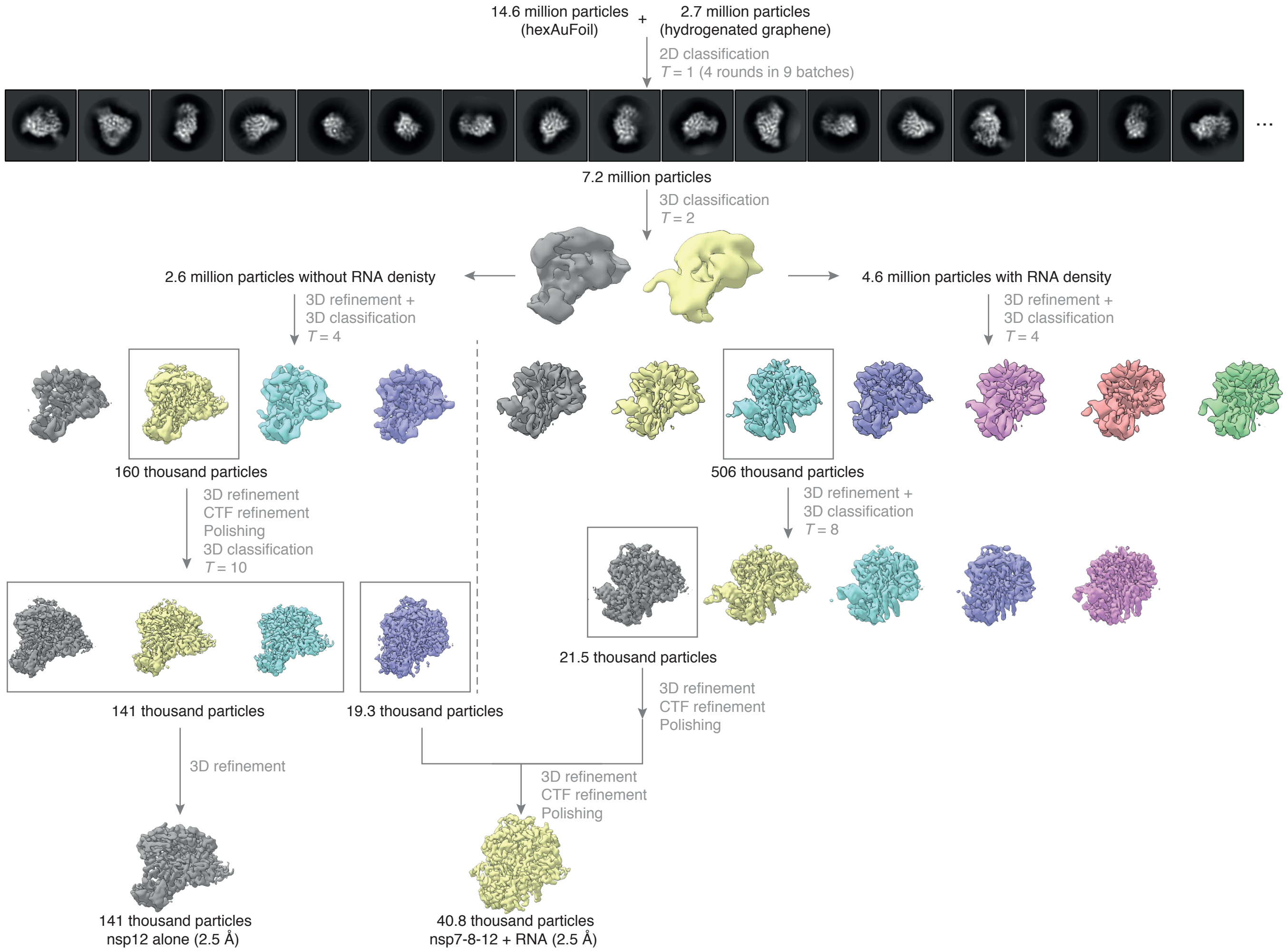

Figure S2: **Cryo-EM data processing summary.** The flowchart shows the main steps in the data processing, from particle picking, through classification, to final maps. A selected subset of the initial reference-free 2D class averages and all the intermediate 3D class averages computed during the processing of this dataset are shown. All 3D class averages, selected for subsequent rounds of processing, are boxed in gray, and the number of particles in each of these is shown. Further attempts to process the discarded classes are omitted from this chart for clarity, as these data did not contribute to the final particle set.

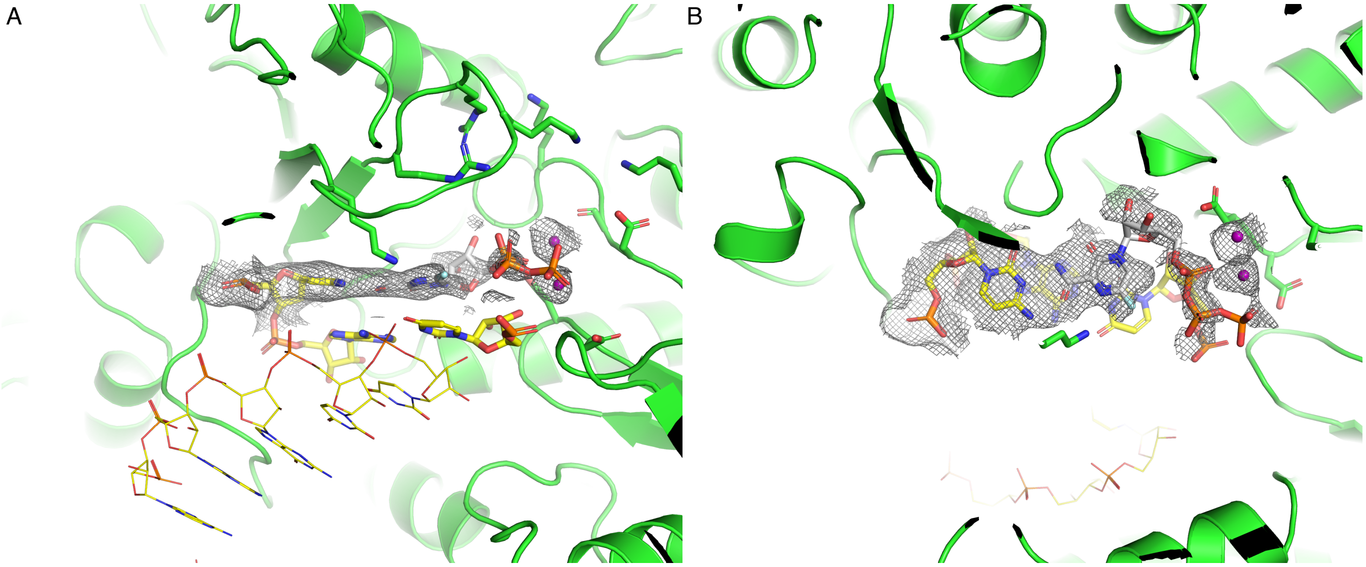

Figure S3: **CryoEM density around the catalytic site of the SARS-CoV-2 RdRp suggests a non-productive binding mode of Favipiravir-RTP.** Two orthogonal views of the polymerase active site, with map density contoured around favipiravir-RTP and Mg^{2+} (**A**-**B**) are shown.

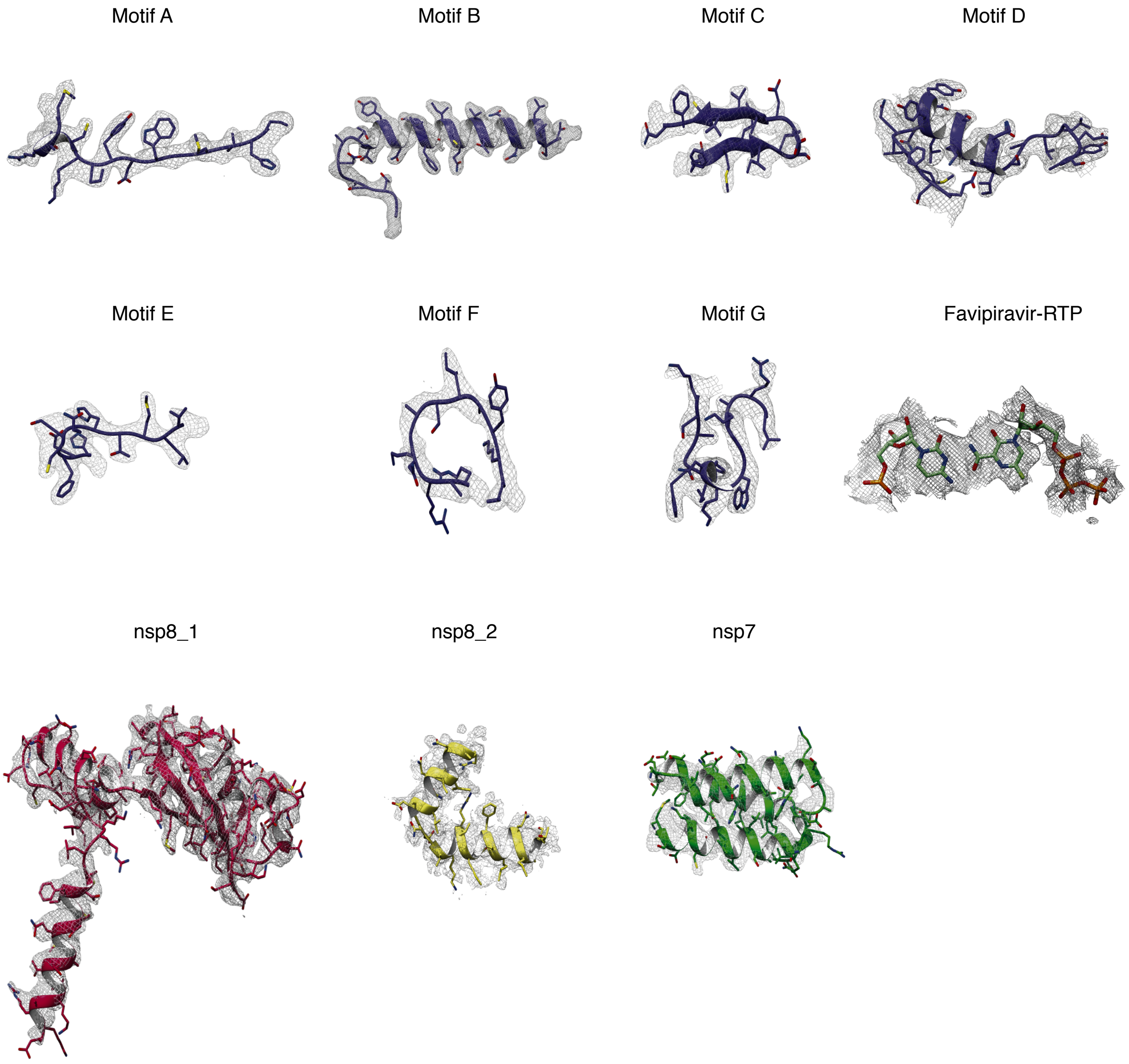

Figure S4: **Map quality and model fitting.** Map density corresponding to the indicated catalytic motifs of the nsp12 subunit, cytosine:favipiravir-RTP, and nsp8_1, nsp8_2, and nsp7 subunits, contoured around the structure of the SARS-CoV-2 RdRp:dsRNA:favipiravir-RTP complex.

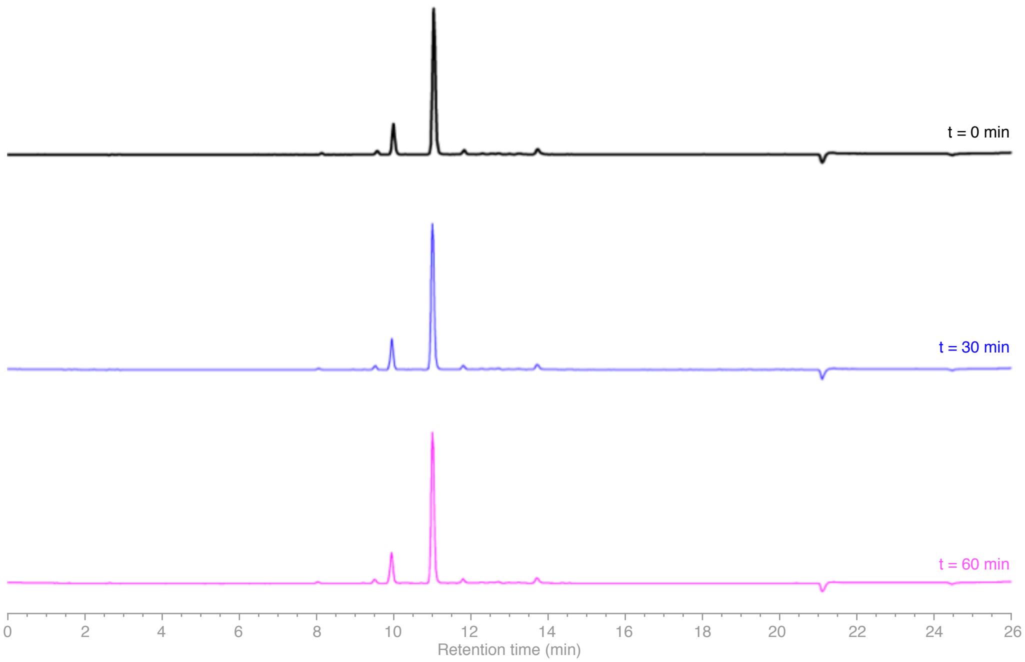

Figure S5: **HPLC stability assay of T-705-RTP.** The stability of T-705-RTP (0.5 mM) under enzymatic assay conditions was monitored using HPLC, via UV absorbance at 360 nm. The solution was incubated at 20°C and was injected into the HPLC device the indicated certain time points. No obvious changes in the peaks were observed after 1 hour.

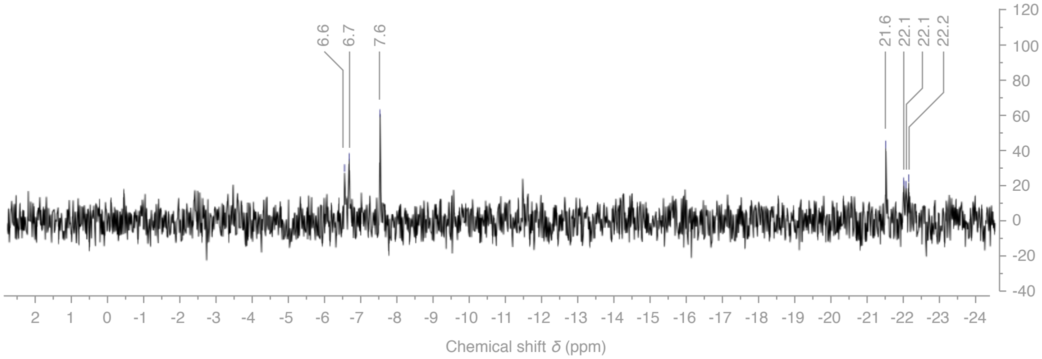

Figure S6: **^{31}P-NMR spectrum of T-705-RTP (0.5 mM) in D2O/H2O = 9:1 containing 1 mM Tris buffer at pH 7.5.** The doublet at $\delta = -6.7$ ppm was assigned to one of the α- or γ-phosphates of the triphosphate moiety, the other expected doublet might have been broadened by chelation to traces of paramagnetic metal ions. The triplet at $\delta = -22.1$ ppm was assigned to the β-phosphate of the triphosphate moiety. The singlet with $\delta = -7.6$ ppm is assigned to inorganic pyrophosphate, a potential contaminant associated with the process of synthesizing T-705-RTP. The singlet with $\delta = -21.6$ ppm is tentatively assigned to cyclic trimetaphosphate.

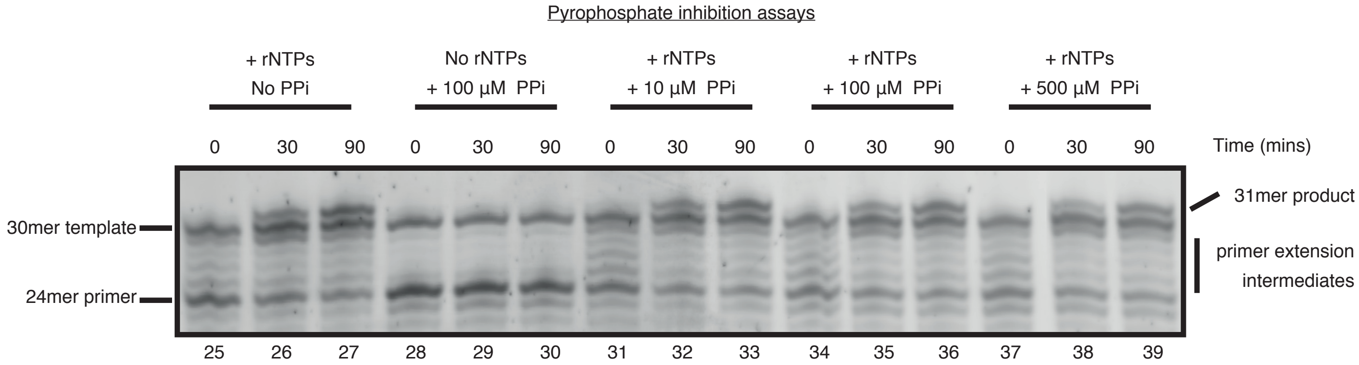

Figure S7: **Pyrophosphate inhibition assays of the RNA-dependent RNA polymerase.** Primer extension assays by the SARS-CoV-2 RdRp were performed in the presence or absence of rNTPs and/or inorganic pyrophosphate (PPi), and RNA products were resolved by denaturing acrylamide gel electrophoresis and visualized by SYBR Gold staining. The presence of pyrophosphate at concentrations between 10 and 500 µM does not affect the RNA replication by the RdRp.

Table S1: **Data, refinement and model statistics.**

**Data collection**	
Microscope	Titan Krios G3i
Energy	300 keV
Camera	Falcon 4
Software	EPU 2.6 (AFIS mode*)
Number of collected micrographs	63,977
Number of micrographs in final reconstruction	17,524
Number of picked particles	14,615,798
Number of particles in final reconstruction	40,828
Nominal magnification	120,000×
Pixel size	0.65 Å
Defocus range	0.3−1.9 µm
Mean defocus	1.1 µm
Electron flux	3.55 $e^-/Å^2/s$
Exposure time	7 s
Electron fluence per frame	1.03 $e^-/Å^2$
Total electron fluence	24.7 $e^-/Å^2$
Number of frames per exposure	24
Specimen temperature	≃80K
Particle box size	$(320\ px)^3$
Resolution (0.143 FSC, masked)	2.5 Å
Efficiency of orientation distribution	0.68
Map sharpening B-factor	20 $Å^2$
**Model composition**	
Non-hydrogen atoms	9,400
Amino acid residues	1,120
Nucleotides	21
**Refinement**	
Resolution range	107.9 - 2.5 Å
Fourier shell correlation (map vs. refined model at 0.5)	2.6 Å
Average Fourier shell correlation	0.86
**RMS deviations**	
Bonds	0.014 Å
Angles	1.56°
**Validation**	
Molprobity score	1.16
Clashscore, all atoms	3.74
Favored rotamers	99.7%
Poor rotamers	0%
Cβ deviations (>0.25 Å)	0%
Ramachandran favored	98.5%
Ramachandran outliers	0%

* AFIS is the "Aberration-Free Image Shift" mode of beam shift and image acquisition.
